# Trophic Relationships of Aquatic Species Offer Valuable Insights Into Shallow Lake Ecosystem Recovery

**DOI:** 10.1002/ece3.72416

**Published:** 2025-11-02

**Authors:** Yajun Qiao, Min Pang, Mengyu Lv, Tianzhe Zhu, Wennuo Han, Zhao Li, Xiaolong Lin, Peidong Su, Pei Qu, Chunhui Zhang

**Affiliations:** ^1^ School of Chemical & Environmental Engineering China University of Mining & Technology Beijing China; ^2^ Ecology and Environment Bureau of Xiong'an New Area Xiong'an New Area Hebei Province China; ^3^ Marine Ecology Research Center, First Institute of Oceanography Ministry of Natural Resources of the People's Republic of China Qingdao Shandong Province China; ^4^ Ecological and Environmental Monitoring Center of Xiong'an New Area Xiong'an New Area Hebei Province China; ^5^ China National Environmental Monitoring Center Beijing China; ^6^ College of Fisheries and Life Science Shanghai Ocean University Shanghai China

**Keywords:** ecosystem restoration, energy flow, shallow freshwater lake, stable isotopic analysis, trophic structure

## Abstract

Shallow freshwater lakes are vital for ecological and climate regulation, but many are degrading. Although restoration efforts are underway to improve water quality, research on their trophic structures is still limited. Baiyangdian Lake (BYD), a typical shallow lake in northern China, has seen improvements in water quality, biodiversity, and aquatic plant biomass after restoration. However, it remains unclear whether its food web's trophic structure has fully recovered. This study collected various field samples from BYD in 2023 and reconstructed BYD's current trophic structure using δ^15^N and δ^13^C stable isotopic analysis. Results indicate that plankton constituted the predominant food source for fish and invertebrates, contributing over 70%. Aquatic plants and macroalgae contributed more significantly to humus and soil organic matter in the sediment than to the main food web. Despite the high biomass of aquatic plants, trophic relationships among fauna in BYD are predominantly reliant on pelagic‐based energy flow pathways. This suggests a potential hysteresis response of its food web structure and ecosystem function to environmental restoration efforts. This study highlights the underestimated long‐term ecological impacts of human activities and emphasizes the need for extended restoration to re‐establish trophic relationships and restore ecosystem health.

## Graphical abstract

Trophic relationships indicated a hysteretic food web response to restoration. Plankton was the primary food source for fish and invertebrates in BYD. Aquatic plants primarily contributed to the humus formation in BYD.

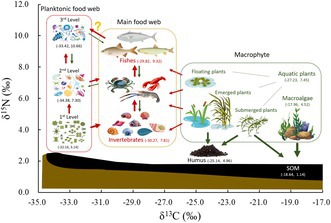

## 1 Introduction

Freshwater lakes, which cover approximately 4% of the Earth's non‐glaciated land surface (Verpoorter et al. 2014), are essential for both natural and human well‐being. They provide critical ecological services to surrounding communities, including water supply, climate regulation, improvement of regional environmental quality, provision of biological habitats, and preservation of biodiversity (Vasistha and Ganguly 2020; Poikane et al. 2024). Furthermore, lakes play a pivotal role in promoting sustainable economic development and maintaining ecosystem functionality (Post 2002). However, the growing demand for natural resources has resulted in widespread degradation of freshwater lake ecosystems. These ecosystems are among the most altered environments globally due to pressures such as nutrient pollution from agriculture and wastewater, climate change, hydrological alterations, invasive species, and overfishing (Poikane et al. 2024; Jenny et al. 2020; Søndergaard et al. 2007).

Many restoration approaches have been implemented for lake ecosystem recovery, such as nutrient load reduction, water levels regulation, and hydrological management (Poikane et al. 2024). These methods, involving either direct or indirect intervention in the ecosystem, contribute to water quality improvement, which in turn enhances biodiversity and provides additional social benefits. For instance, improved water quality increases safety for local communities that rely on shallow lakes for drinking water, recreation, and fish production. Consequently, water quality has become a widely used indicator for assessing restoration success in freshwater shallow lakes (Dondajewska et al. 2019; Tammeorg et al. 2024). However, shallow lake ecosystems are complex systems comprising numerous biotic and abiotic components. Restoration effectiveness in shallow lakes can be evaluated not only through environmental indicators such as water quality but also by examining whether trophic relationships within the food web are reasonable, stable, and sustainable. Trophic structure, a network of multiple species and their trophic relationships, describes the complex nutrient interactions between different species within a biological community and reveals the processes of material cycling and energy flow at the ecosystem level (Post et al. 2000; Kuiper et al. 2015). Different species occupy distinct trophic positions or trophic levels (TL) within local trophic structures. Trophic relationships are crucial connections among species in a community, providing the basis for survival and indicating the energy flow within an ecosystem (Rooney and McCann 2012). In recent years, many studies have focused on exploring the trophic structure of various aquatic ecosystems to better understand ecosystem structure and function. Particular attention has been given to lake ecosystems, where extensive research has been carried out to investigate their trophic dynamics for the purpose of conserving biodiversity and safeguarding aquatic environments. Stable isotope analysis (SIA) has been extensively used in the study of aquatic ecosystems for guiding environmental protection, supporting restoration initiatives, and enhancing biodiversity (Li et al. 2023). Previous studies have employed stable isotope analysis to investigate trophic structures in shallow lake ecosystems influenced by various environmental factors. For example, research on Lake Erie in the USA has examined how spatiotemporal heterogeneity affects trophic level (TL) variation through δ^15^N and δ^13^C analyses of primary producers (Guzzo et al. 2011). In North American north‐temperate lakes, combined dietary and isotopic data from multiple fish populations have revealed the role of fish in linking benthic and pelagic food webs (Vander Zanden and Vadeboncoeur 2002). In China, studies on lakes such as Donghu and Chaohu Lakes have shown that external nutrient inputs can influence stable isotope signatures in macrophytes, offering insights into the trophic structure of these freshwater systems (Xu, Li, and Xie 2005; Xu, Xie, et al. 2005). However, research focusing on trophic structures that reflect comprehensive ecological restoration outcomes remains limited. Further research is necessary to fully understand the complexity of trophic interactions and their implications for ecological restoration, particularly in the context of ongoing environmental changes and restoration initiatives.

Baiyangdian Lake (BYD), located in the central plain of Hebei Province, is a representative temperate shallow freshwater lake in northern China, with its primary water sources being upstream river floods and natural precipitation. BYD provides a wide range of ecological services, including water purification and supply management, biodiversity conservation, the maintenance of overall ecological equilibrium, and regulation of the regional climate across the North China region (Tong et al. 2021). Since the 1960s, the ecological environment surrounding BYD has undergone severe degradation due to both natural disasters and human activities. This degradation has resulted in a substantial reduction in water flow, leading to frequent occurrences of dry lake phenomena. Furthermore, aquaculture and agricultural runoff have introduced significant pollutants from upstream sources, exacerbating eutrophication. Local residents' daily activities have further contributed to pollution levels. Consequently, the food web has been negatively impacted, leading to a substantial decline in biodiversity, reduced fish species abundance, and plankton hypermorphosis. These changes have disrupted trophic structures and diminished the provision of ecosystem services (Mao et al. 2023). Since 2017, comprehensive management projects have been implemented for BYD. Many studies have evaluated the restoration effectiveness based on the enhanced biomass of aquatic plants and significantly improved water quality (Zeng et al. 2021; Mao, Zhao, et al. 2024), and the ecological state of BYD has also been assessed using model analysis based on historical survey data (Mao et al. 2023; Zeng et al. 2021). However, as mentioned above, while multiple species inhabit the lake, it remains unclear whether the trophic structure within the food web of BYD has been restored following the restoration efforts.

This study involved systematic and comprehensive sampling of a diverse range of aquatic organisms from BYD, as well as an analysis of their trophic relationships based on δ^15^N and δ^13^C signatures. The objectives of this study were twofold: (1) to assess the current trophic structure of BYD by examining fluctuations in δ^15^N and δ^13^C values among different functional groups, such as primary producers and consumers; and (2) to estimate the TLs of consumers and the contribution proportions of various primary food sources applying MixSIAR models, thereby clarifying the trophic relationships and structure within the shallow lake ecosystem and evaluating whether the biodiversity within BYD has reached a sustainable and healthy state following a series of ecological restoration efforts. The findings will provide a foundational understanding of material cycling and energy flow in shallow freshwater lakes after a series of recovery projects, ultimately supporting the development of effective strategies for their conservation and ecological restoration.

## 2 Materials and Methods

### 2.1 Sample Collection

**FIGURE 1:**
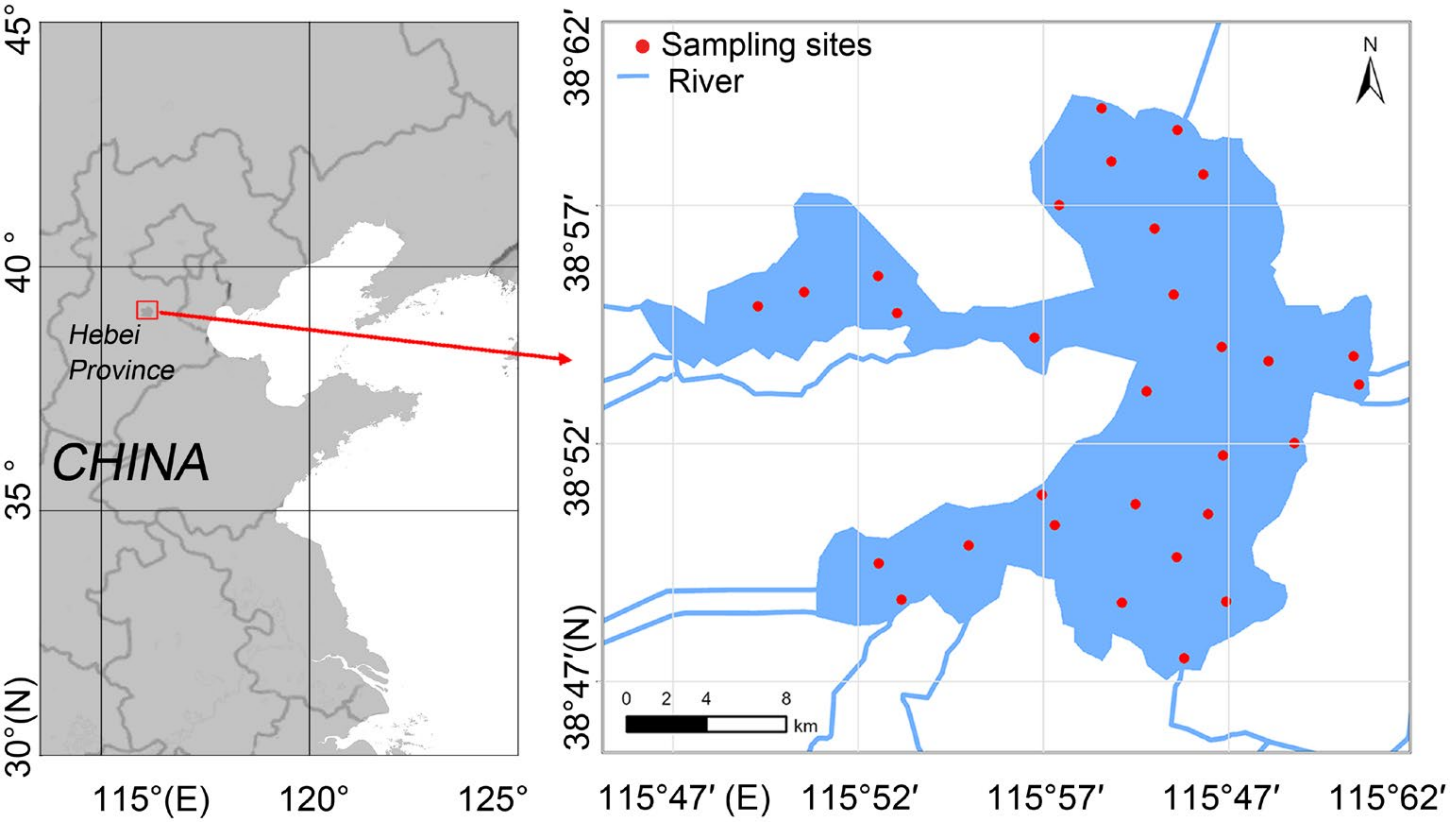
Field sampling sites (●) in Baiyangdian (BYD) lake.

To investigate BYD food web dynamics, a cruise was conducted in May 2023, and samples were systematically collected from designated sites within the area, as shown in Figure 1. Fish and invertebrate specimens were collected from each location using horizontal midwater and bottom trawling methods (Olivar et al. 2012). Onboard, the specimens were identified, meticulously documented with photographs, and then stored at −20°C for subsequent stable isotope analysis. Potential food sources, including plankton, aquatic plants (emerged, floating, and submerged species), and macroalgae, were also collected. Mixed plankton samples were collected by filtering 100 L of water through a No. 25 nylon mesh plankton net. For samples associated with aquatic plants, collection methods varied: free‐floating species were captured using the No. 25 plankton net, whereas submerged or emergent species were manually harvested using grabs, sickles, and trowels. It should be noted that certain aquatic carnivorous plants, such as *Utricularia vulgaris* , have evolved specialized bladder traps—modified leave structures that capture microfauna to supplement their nitrogen intake. These bladder traps were specifically identified and excluded from sampling to ensure data accuracy and study reproducibility. Sediment samples were obtained using a bucket dredge in the subtidal zone.

### 2.2 Sample Pretreatment and Stable Isotope Analyses

The decision to analyze samples partially or in their entirety was based on the feeding habits of consumers. Mixed plankton samples, comprising individuals from multiple species, were collected together, while the fronds of aquatic plants were gathered for analysis. The tissues (e.g., muscles) of fish and invertebrates provide long‐term information on food selection (McIntyre and Flecker 2006). Therefore, the muscles of fish and invertebrates were specifically extracted and analyzed.

A pretreatment procedure based on a previous study (Qu et al. 2016) was refined. Specimens were divided into smaller segments, dried in an oven at 80°C for a minimum of 48 h until achieving a constant weight, and then ground into a fine powder using a mortar and pestle. Considering that acidification can eliminate carbonates and affect δ^15^N values in dietary sources, each sample was divided into two portions: one portion remained untreated for direct δ^15^N analysis, while the other portion was treated with 1 mol/L hydrochloric acid to remove carbonates for δ^13^C analysis.

Following pretreatment, the N and C isotope ratios were determined using continuous flow isotope ratio mass spectrometry with a Thermo Delta Q isotope ratio mass spectrometer (Thermo Electron, Bremen, Germany). The values of δ^15^N and δ^13^C were defined as:

(1) δN15‰=N15/Nsample14N15/Nair15−1×1000

(2) δC13‰=C13/Csample12C13/CVPDB12−1×1000

In the formulae, the ratios of heavy isotopes to light isotopes in the samples are represented by ^15^N/^14^N_sample_ and ^13^C/^12^Csample. The ^15^N/^14^N_air_ ratio indicates the atmospheric N_2_ standard for ^15^N, whereas the ^13^C/^12^C_VPDB_ ratio corresponds to the Vienna Pee Dee Belemnite (VPDB) standard for ^13^C.

### 2.3 Food Source Analysis

In this study, plankton, aquatic plants, and macroalgae were identified as the primary food sources for local organisms and the main energy contributors to the trophic structure. When the number of food sources in a food web is limited, the MixSIAR model was employed to estimate the proportional contribution of each source to the consumer's diet. This model is based on a Bayesian framework and incorporates Dirichlet Allocation, enabling it to accurately quantify the proportion of prey in the predator's diet (Fry 2013).

### 2.4 TL Analysis

N isotope ratios typically display a consistent increase along the food chain, with noticeable transfer occurring at each successive TL (Smit et al. 2005). The variations between different TLs can be attributed to N isotopic fractionation, leading to ^15^N enrichment through the food chain due to consumer ingestion and metabolic processes (Caut et al. 2010). This phenomenon enables the determination of TLs using a model formula that incorporates a recognized trophic fractionation factor of 3.4‰ for δ^15^N (Δ^15^N) between adjacent TLs (Post 2002):

(3) $$\mathrm{TL}=\frac{\delta^{15}N_{c}-\delta^{15}N_{b}}{\Delta^{15}N}+{\mathrm{TL}}_{b}$$

where δ^15^N_c_ is the N isotope ratio of the consumer and Δ^15^N is the trophic fractionation factor mentioned above (3.4‰). In this study, δ^15^N_b_ served as the baseline for TL calculation. Specifically, zooplankton was selected as the baseline organism for pelagic species, while the snail *Cipangopaludina chinensis* was designated as the baseline organism for benthic organisms, as described by Zhang et al. (2024). TL_b_ represents the TL of the primary consumer relative to a specific δ^15^N_b_. In this study, the value of TL_b_ was set to 2.

### 2.5 Statistical Analysis

All statistical analyses were conducted using SPSS 17.0 software (SPSS Inc., Chicago, IL, USA). To examine variations in stable isotope signatures among different sample types, one‐way analysis of variance (ANOVA) was performed on δ^15^N and δ^13^C data across the following groups: fish, invertebrates, aquatic plants, plankton, macroalgae, humus, and sedimentary organic matter (SOM). When statistically significant differences were observed (*p* < 0.05), a *t*‐test was applied for pairwise comparisons to determine which specific groups differed significantly.

## 3 Results

### 3.1 δ^15^N and δ^13^C Stable Isotope Characteristics of Samples From BYD

To explore the stable isotope characteristics of different components within the BYD ecosystem, 226 samples were collected from 41 representative species across the main food web, involving 7 species of macroinvertebrates, 13 species of fish, 18 species of aquatic plants, 1 species of macroalgae, and mixed plankton samples (Table S1). Additionally, humus and SOM were collected as environmental reference materials. All samples were analyzed for δ^15^N and δ^13^C values, and one‐way ANOVA was conducted to assess whether significant differences existed in δ^15^N and δ^13^C among different sample types (SOM, humus, plankton, macroalgae, aquatic plants, invertebrates, fish). As shown in Figure 1, fish in BYD had δ^15^N values ranging from 6.81‰ to 14.41‰ (mean: 9.32‰), and δ^13^C values ranging from −33.23‰ to −24.29‰ (mean: −29.82‰). Invertebrates had δ^15^N values from 5.78‰ to 11.28‰ (mean: 7.81‰), and δ^13^C values from −33.54‰ to −27.10‰ (mean: −30.72‰). Aquatic plants showed a wide range of δ^15^N values, from 1.43‰ to 12.20‰ (mean: 7.15‰), and a particularly wide range of δ^13^C values from −35.12‰ to −15.28‰ (mean: −25.90‰). Plankton also exhibited a wide range of δ^15^N values, from 2.17‰ to 12.04‰ (mean: 7.18‰), and δ^13^C values from −37.17‰ to −29.97‰ (mean: −33.56‰). The δ^15^N and δ^13^C values for macroalgae ranged from 4.21‰ to 4.77‰ and −19.31‰ to −16.71‰, respectively. The δ^15^N values of SOM and humus were from 0.38‰ to 2.09‰ and from 3.65‰ to 6.14‰, respectively, with δ^13^C values ranging from −21.99‰ to −13.91‰ and from −30.67‰ to −18.19‰, respectively.

The δ^15^N values of fish were significantly higher than that of invertebrates, aquatic plants, plankton, humus, macroalgae, and SOM (*p* < 0.01). The δ^15^N values of invertebrates were significantly higher than those of humus, macroalgae, and SOM (*p* < 0.01), indicating a discernible TL difference. The δ^15^N range of aquatic plants was relatively broad (1.43‰–12.20‰), partially overlapping with the δ^15^N ranges of plankton (2.17‰–12.04‰) and invertebrates (5.78‰–11.28‰). However, their mean values did not differ significantly (*p* > 0.05), suggesting potential complexity in material sources or trophic relationships among these groups.

**FIGURE 2:**
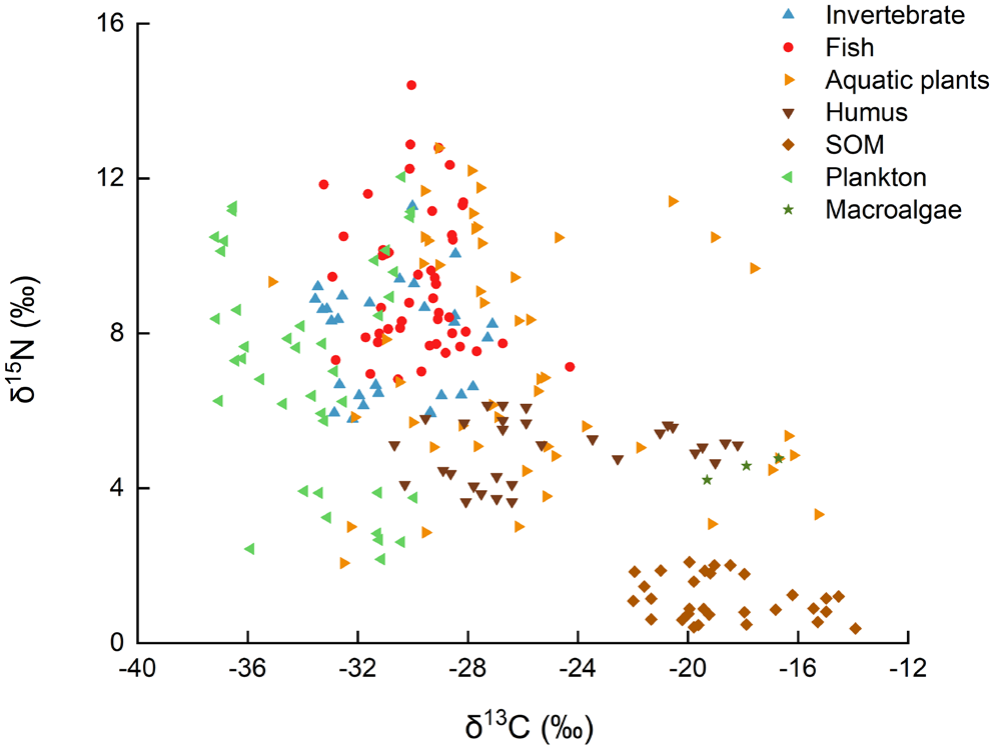
Dot plot showing the δ^13^C and δ^15^N values of aquatic organisms from Baiyangdian Lake. SOM, sedimentary organic matter.

Significant differences in δ^13^C values were also observed across sample types. Plankton exhibited the lowest mean δ^13^C values (−33.56‰ ± 2.42‰), which were significantly lower than those of other sample types (*p* < 0.05), potentially reflecting the distinct carbon source characteristics of plankton in the aquatic environment. Macroalgae had a mean δ^13^C value of −17.96‰ ± 1.06‰, significantly higher than those of plankton, invertebrates, aquatic plants, fish, and humus (*p* < 0.05), indicating a unique carbon fixation pathway or habitat‐specific carbon source. The δ^13^C values of fish (−29.82‰ ± 1.66‰) were significantly higher than those of plankton and invertebrates (*p* < 0.05), but not significantly different from those of aquatic plants (*p* > 0.05) (Figure 2).

### 3.2 Trophic Levels of Aquatic Fauna in BYD

**FIGURE 3:**
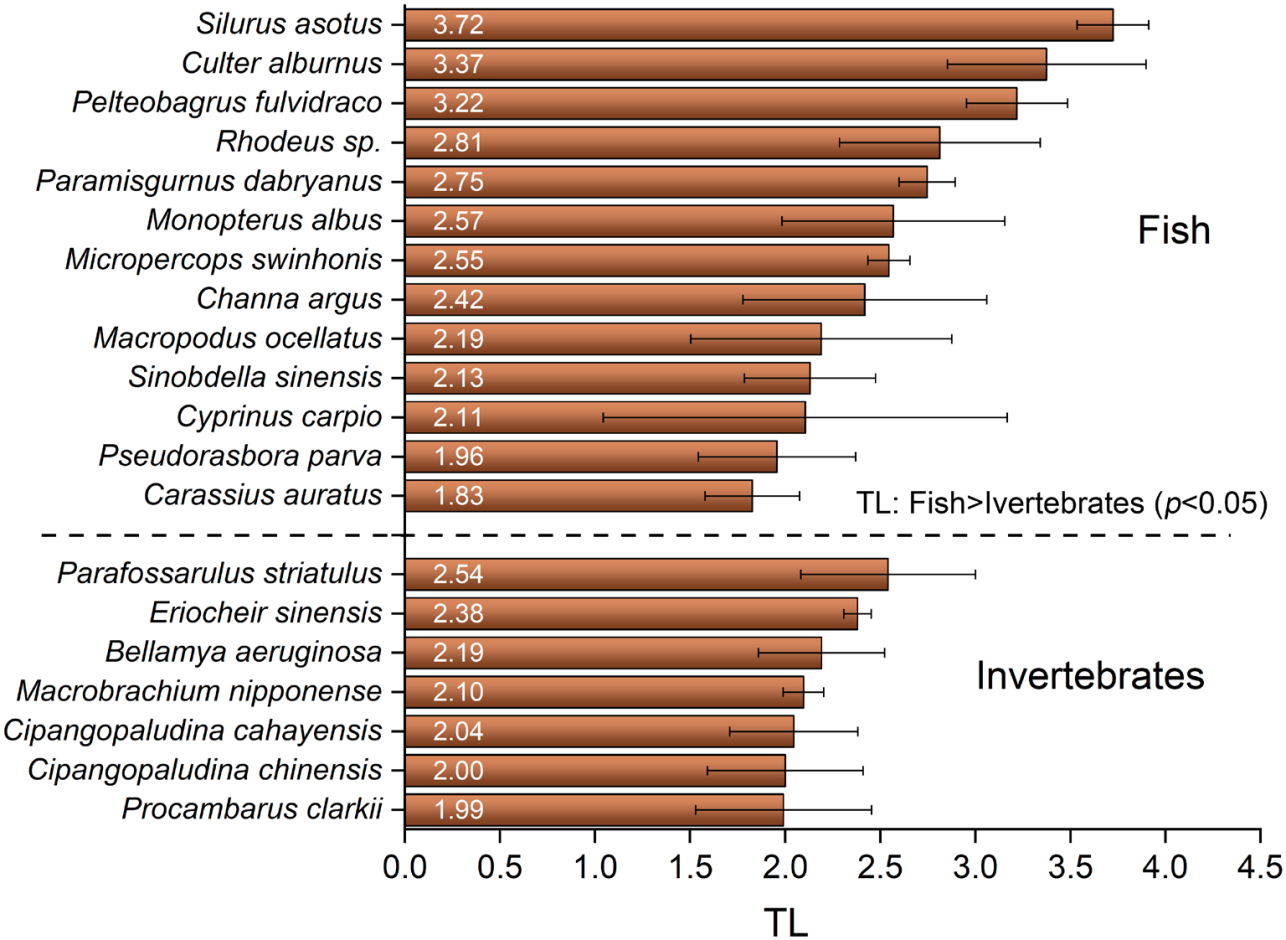
The trophic levels of 13 primary fish species (*n* = 47) and 7 invertebrate species (*n* = 29) in Baiyangdian Lake are presented. Error bars represent the standard deviation of trophic levels (TLs) among individuals within each species. A *t*‐test analysis indicated that, collectively, fish species occupied significantly higher trophic levels than invertebrate species.

To accurately determine the TL of aquatic fauna, the baseline for TL calculation was established by considering the contributions of the three primary food sources mentioned above. As shown in Figure 3, the mean TLs of fish and invertebrates ranged from 1.83 to 3.72, while those of invertebrates ranged from 1.99 to 2.54. Among the fish species, *Silurus asotus, Culter alburnus*, and *Pelteobagrus fulvidraco* exhibited the highest TLs, with mean values of 3.72, 3.37, and 3.22, respectively. Among invertebrates, *Parafossarulus striatulus*, *Eriocheir sinensis* , and *Bellamya aeruginosa* had the highest mean TL values of 2.54, 2.38, and 2.19, respectively. The statistically significant higher mean TLs were observed in fish compared to invertebrates (*p* < 0.05).

### 3.3 Contribution of Potential Food Sources for Aquatic Fauna in BYD

**FIGURE 4:**
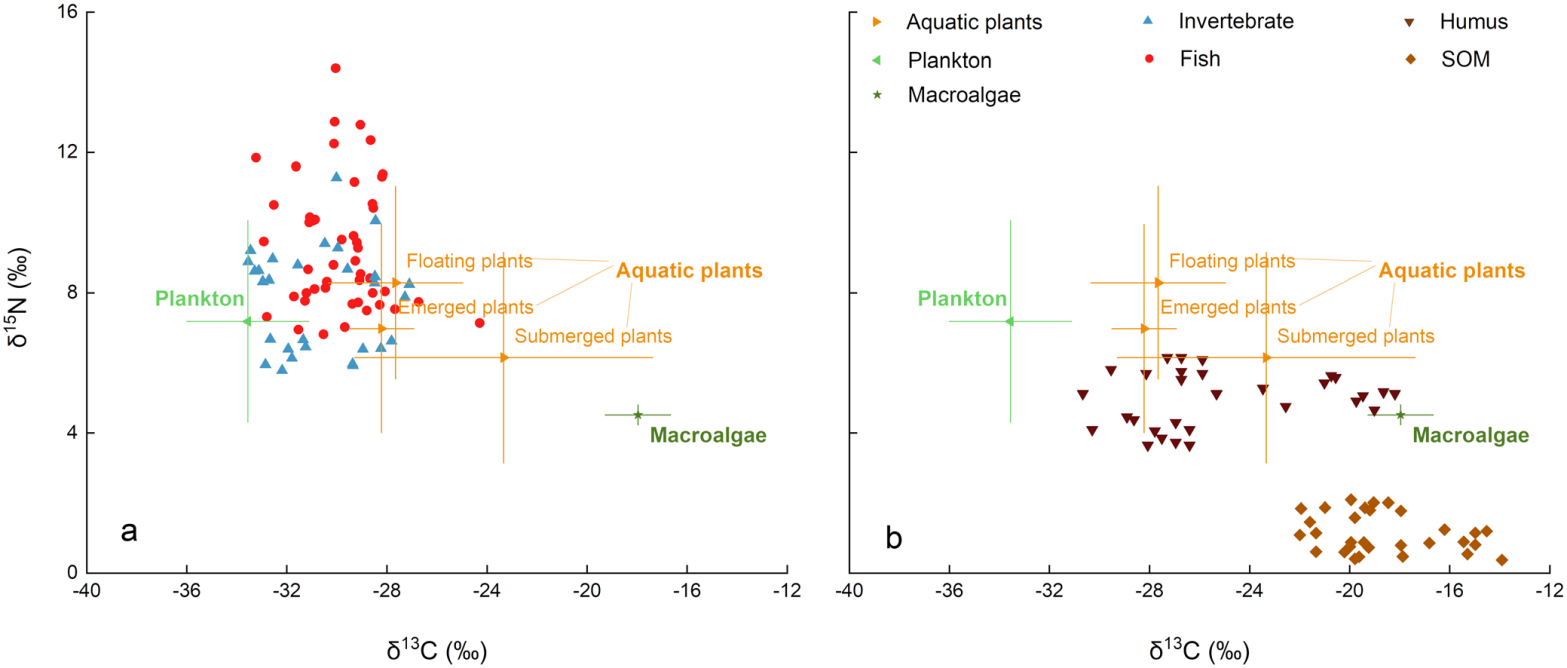
δ^13^C and δ^15^N distribution patterns of the main food sources (plankton, aquatic plants, and macroalgae) compared to fish (●), invertebrates (▲), humus (▼), and SOM (♦). SOM, sedimentary organic matter.

**FIGURE 5:**
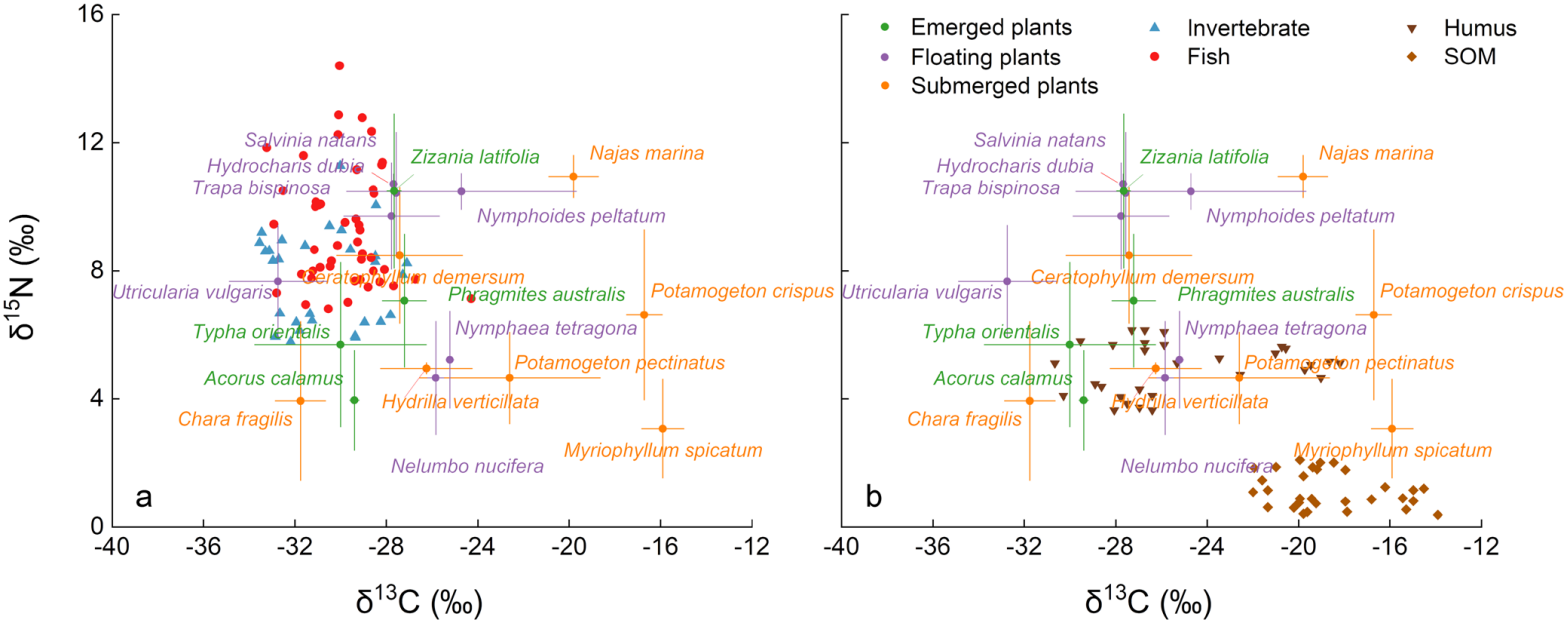
δ^13^C and δ^15^N distribution patterns of aquatic plants, including emerged, floating, and submerged species in BYD compared to fish (●), invertebrates (▲), humus (▼), and SOM (♦). SOM, sedimentary organic matter.

Within the study area, three primary producers, including mixed plankton, aquatic plants, and macroalgae (*Ulotrichaceae* sp.), were identified as main food sources due to their amounts. The δ^13^C and δ^15^N distribution patterns of the main food sources (plankton, aquatic plants, and macroalgae) were shown in Figure 4, compared with those of fish and invertebrates in the main food web (Figure 4a), and with humus and SOM in the sediment (Figure 4b). From Figure 4, it can be observed that the δ^13^C values of plankton and some aquatic plants, including floating and emerged species, overlap with those of fish and invertebrates. In contrast, the δ^13^C values of all aquatic plants and macroalgae coincide with those of humus and SOM. Further analysis of δ^13^C and δ^15^N distributions for specific aquatic plant species was presented in Figure 5. The δ^13^C values of many floating plants, such as *Salvinia natans* , *Utricularia vulgaris* , *Nelumbo nucifera* , *Trapa bispinosa* , and *Hydrocharis dubia*, as well as some emerged plants like *Acorus calamus* , *Typha orientalis*, *Phragmites australis* , and *Zizania latifolia* , coincide with the δ^13^C values of fish and invertebrates. Meanwhile, the δ^13^C values of certain submerged species (including *Hydrilla verticillate*, *Potamogeton pectinatus* , *Najas marina* , *Potamogeton crispus* , and *Myriophyllum spicatum* ), along with floating species (including *Nelumbo nucifera* , *Nymphaea tetragona* , and *Nymphoides peltatum*), and the macroalgae *Ulothrix* sp., coincide with the δ^13^C values of humus and SOM.

**FIGURE 6:**
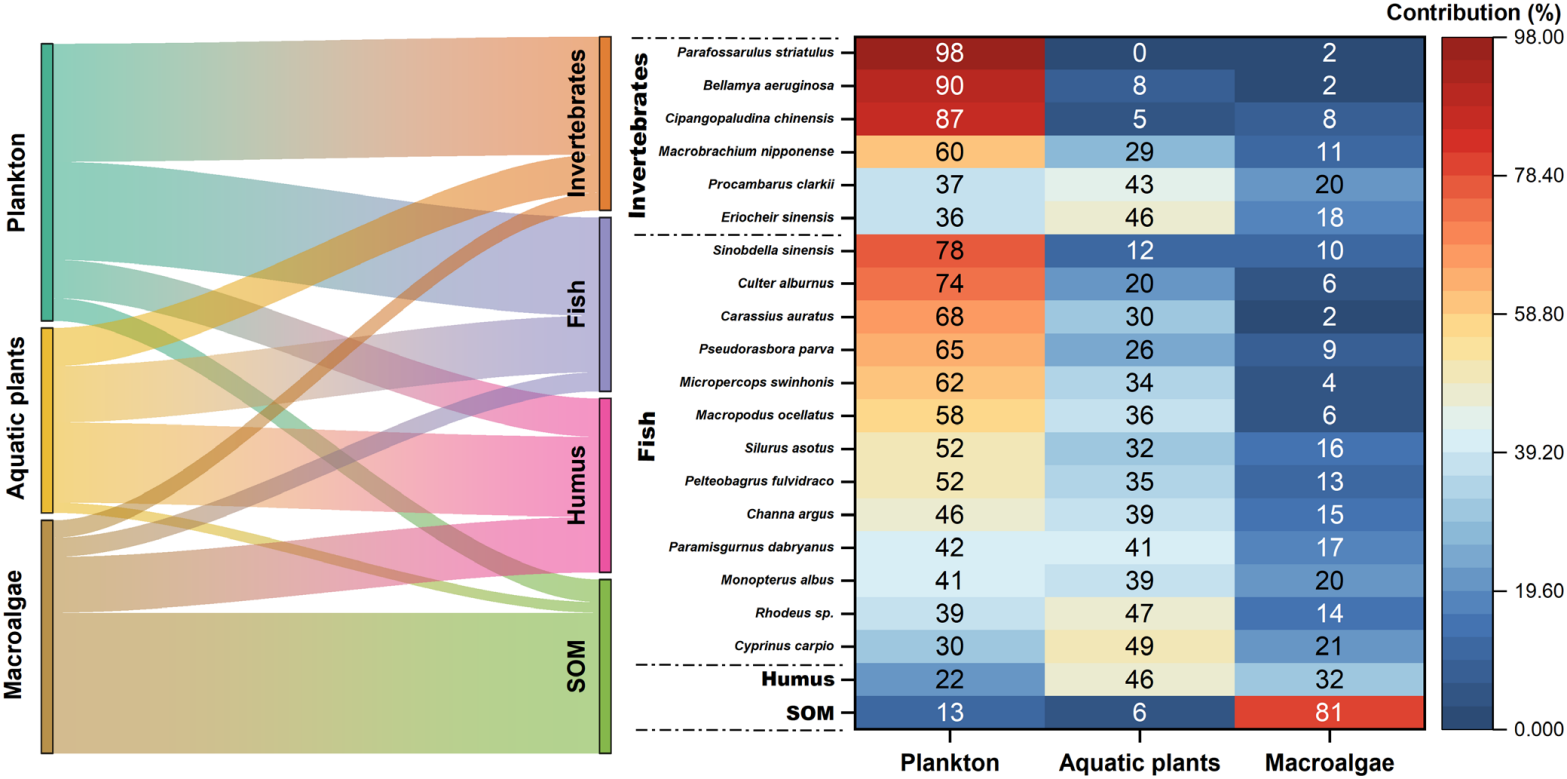
Heat map revealing the relative contributions of plankton, aquatic plants, and macroalgae to the primary fish and invertebrate species in Baiyangdian Lake.

**FIGURE 7:**
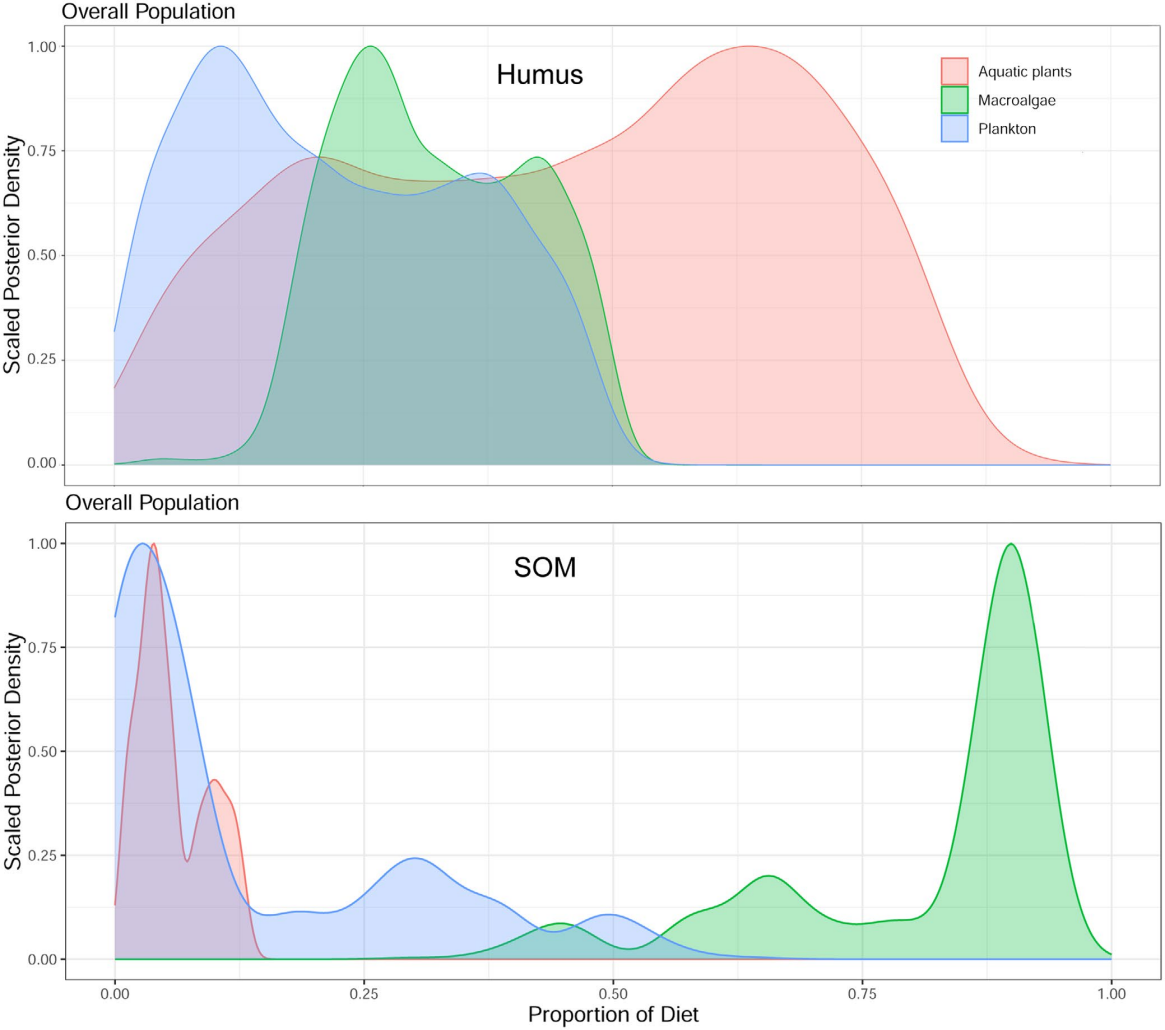
The relative contributions of plankton, aquatic plants, and macroalgae to the humus (upper) and SOM (lower) in Baiyangdian Lake.

The contribution of these three primary producers to invertebrates and fish from BYD was further analyzed using MixSIAR as described above. As shown in Figure 6, for invertebrates, plankton was the primary contributor to aquatic fauna, particularly for snail species such as *Cipangopaludina chinensis* , *Bellamya aeruginosa*, and *Parafossarulus striatulus*, which derived over 80% of their diet from plankton. In contrast, for the invertebrates *Eriocheir sinensis* and *Procambarus clarkii* , aquatic plants were important food sources, contributing 46% and 43.3%, respectively. For fish, plankton was the primary food source, followed by aquatic plants and macroalgae. Plankton contributed over 70% to the diets of *Sinobdella sinensis* and *Culter alburnus* , and more than 60% to those of *Carassius auratus* , *Micropercops swinhonis* , and *Pseudorasbora parva* . It was noteworthy that aquatic plants contributed a significantly higher proportion (47%) compared to plankton (39%) in the diet of *Rhodeus* sp. Humus and SOM were the main carbon sinks in the BYD ecosystem. Macroalgae contributed significantly to SOM, accounting for approximately 98% of its composition. Aquatic plants contributed 48% to the formation of humus, which was significantly higher than the contributions from both macroalgae and plankton (Figure 7).

## 4 Discussion

### 4.1 Trophic Relationship Within Food Web in BYD Post‐ Restoration

Within the BYD trophic relationships revealed in this study, the δ^13^C features of organisms indicate that plankton is the predominant food source for fish and invertebrates, while aquatic plants contribute to a limited extent, and macroalgae play a minimal role in the food web (Figure 4). The contribution of potential food sources for aquatic fauna in BYD presented in Figure 6 also demonstrated this conclusion.

BYD is the largest freshwater wetland system on the North China Plain, distinguished by its significant spatial and temporal diversity. There are over 100 lakes and ponds in this region that are interconnected by an extensive network of rivers, canals, and channels (Wang et al. 2024). Over the past two decades, BYD has been primarily influenced by intensive human activities, including water pollution and water shortage, which have led to a series of problems attributed to water quality deterioration and biodiversity reduction (Zheng et al. 2024). Mao, Liu, et al. (2024) revealed the occurrence of two significant ecological shifts in BYD: from a clear macrophyte‐dominated state to a turbid phytoplankton‐dominated state. The first shift was initiated by dam construction in the basin, which prolonged the hydraulic residence time and consequently accelerated nutrient enrichment around 1963, while the second shift was attributed to the intensification of human activities and a rise in regional temperature during the period from 1990 to 2017 (Mao, Liu, et al. 2024). Plankton, particularly phytoplankton, serves as the main primary producer in lake ecosystems and is widely utilized as a key indicator of water quality (Boyer et al. 2009). Excessive phytoplankton growth, often linked to eutrophication, can disrupt the stability of lake ecosystems (Havens et al. 2019). The transition from macrophyte dominance to phytoplankton dominance alters the original food web structure, affecting energy flows from primary producers to top predators (Chao et al. 2022).

Since the establishment of the Xiong'an New Area, increasing attention has been paid to the maintenance of aquatic ecosystem health to protect and enhance aquatic biodiversity (Mao, Zhao, et al. 2024). A series of policies considering water quality, ecology, and water volume have been implemented to restore the environmental quality of BYD. Key issues such as pollution control, water replenishment, silt removal, and aquatic vegetation reconstruction have been addressed in comprehensive lake management programs (Huang et al. 2024). The recovery of macrophytes has been recognized as a critical ecological strategy for rehabilitating degraded lake ecosystems and enhancing the water quality of shallow eutrophic lakes (Phillips et al. 2016; Hilt et al. 2018). After a series of ecological restoration treatments, the water quality of BYD has shown a substantial improvement in 2023 compared to the period 2018–2020 (P. China Ministry of Ecology and Environment 2023), and the biomass of aquatic plants has also been enhanced in BYD (Mao, Zhao, et al. 2024).

**FIGURE 8:**
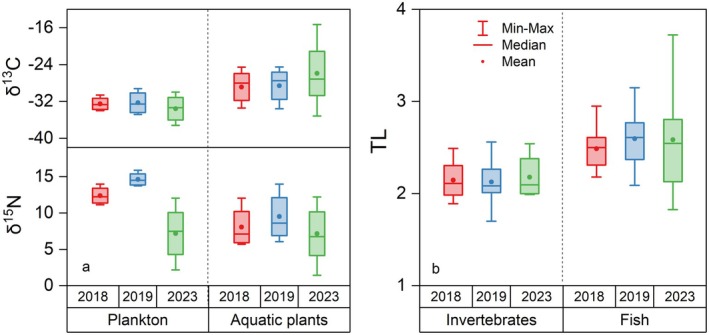
Changes in δ^13^C and δ^15^N values of primary producers (plankton and aquatic plants), along with trophic level (TL) changes of both invertebrates and fish from 2018, 2019 to 2023. Stable isotopic data for BYD fauna in 2018–2019 were obtained from the supporting information of Zhang et al. (2024) study.

There were limited references regarding stable isotopic characteristics in BYD before restoration. Zhang et al. (2024) analyzed the stable isotopic characteristics of representative fauna during 2018 and 2019 within BYD (Tables S2–S4). Compared with the results in Zhang's study, we observed a broader range of δ^13^C values, indicating the cultivation of a greater diversity of aquatic plant species, particularly C4 plants. As shown in Figure 8a, the δ^15^N characteristics of primary producers, especially plankton, in 2023 exhibited significant changes compared to 2019. This may be attributed to nitrogen management practices, including water replenishment and dredging activities, implemented during the restoration process (Zhang et al. 2021).

During the ecological restoration period from 2018 to 2023, the TLs of invertebrates remained stable, suggesting minimal disturbance caused by ecological restoration. In contrast, the TL range of fish increased, reflecting positive restoration outcomes with more fish at higher TL present in BYD (Figure 8b). However, the unchanged mean TL values of fish suggest a relatively higher abundance of species at lower TLs and a scarcity of top predators. This comparison indicates that the restoration of BYD is progressing positively; however, it remains significantly distant from achieving the most optimal restoration outcome.

Besides, the present findings also indicate that the food web structure in BYD post‐ restoration continues to exhibit plankton dominance characteristics. The extensive δ^15^N range observed for plankton indicated a highly dynamic and complex planktonic BYD food web (Figures 2 and 8). Because BYD is a shallow aquatic grass‐type lake, aquatic plants, including emerged, floating, and submerged species, exhibit a high biomass in BYD. However, the findings of this study indicated that aquatic plants, particularly submerged species, contributed relatively less to the main food web (Figure 4). This may be attributed to the relatively low population of herbivorous fish in BYD (Li et al. 2018). Consequently, although a greater diversity of aquatic plant species, especially those C4 plants as shown in Figure 8, have been planted in BYD, only a minor portion of these aquatic plants served as a food source for higher TLs. Instead, they played a more substantial role in humus formation (48%) (Figures 6 and 7), which either re‐enters the ecosystem via the detrital food chain or accumulates on the lakebed. Due to the δ^13^C and δ^15^N distributions of aquatic plant species shown in Figure 5, certain submerged species (including *Hydrilla verticillate*, *Potamogeton pectinatus* , *Najas marina* , *Potamogeton crispus* , and *Myriophyllum spicatum* ) and floating species (including *Nelumbo nucifera* , *Nymphaea tetragona* , and *Nymphoides peltatum*) did not directly participate in the main food web, but instead contributed to the formation of humus and SOM. Notably, the macroalgae *Ulothrix* sp., which forms filamentous patches loosely attached to the bottom, contributed 94% of the organic matter, despite its minor role in the food web. These results were consistent with the findings of previous studies showing that detritus derived from dead macrophytes contribute minimally to the diets of consumers at higher TLs (Mao et al. 2021). The presence of lignin and robust cell walls might inhibit the assimilation of decomposed macrophytes as a carbon source.

### 4.2 Ecological Restoration Assessment Based on Trophic Structure Within Food Webs

As discussed above, the increased diversity of aquatic plant species and the expanded TL range of fish within BYD indicated that the restoration is progressing positively; however, it has yet to achieve the most optimal restoration outcomes. The current trophic structure in BYD remained heavily reliant on plankton. Macroalgae were identified as a significant contributor to SOM, while approximately half of the aquatic plants contributed to humus formation (Figures 6 and 7). Both macroalgae and aquatic plants played relatively minor roles in supporting the primary food webs in BYD. Despite improvements in water quality within BYD, the restoration of its food web structure and ecosystem function did not progress concurrently. The current contributions of various food sources to the trophic structure within the main food web of BYD were consistent with a recent study using ECOPATH steady‐state food web models and historical data, which revealed that changes in food web structure and ecosystem function driven by hydrological variations in Hulun Lake, northern China, exhibited potential hysteresis (Xue et al. 2025). These results suggest that the profound impact of environmental changes on food web structure might be difficult to restore in a short time period.

Biological communities are an essential component of aquatic ecosystems, serving as both indicators of environmental change and key factors in evaluating the functionality and stability of lake ecosystems. In addition to species composition and community structure, trophic relationships in food webs provide information for network representation of feeding interactions and energy flow distribution within an ecosystem (Xu, Ji, et al. 2016). Therefore, the analysis of trophic relationships in food webs can provide critical insights into ecosystem stability and health, the ultimate goals for the management of most lakes (Kuiper et al. 2015).

**FIGURE 9:**
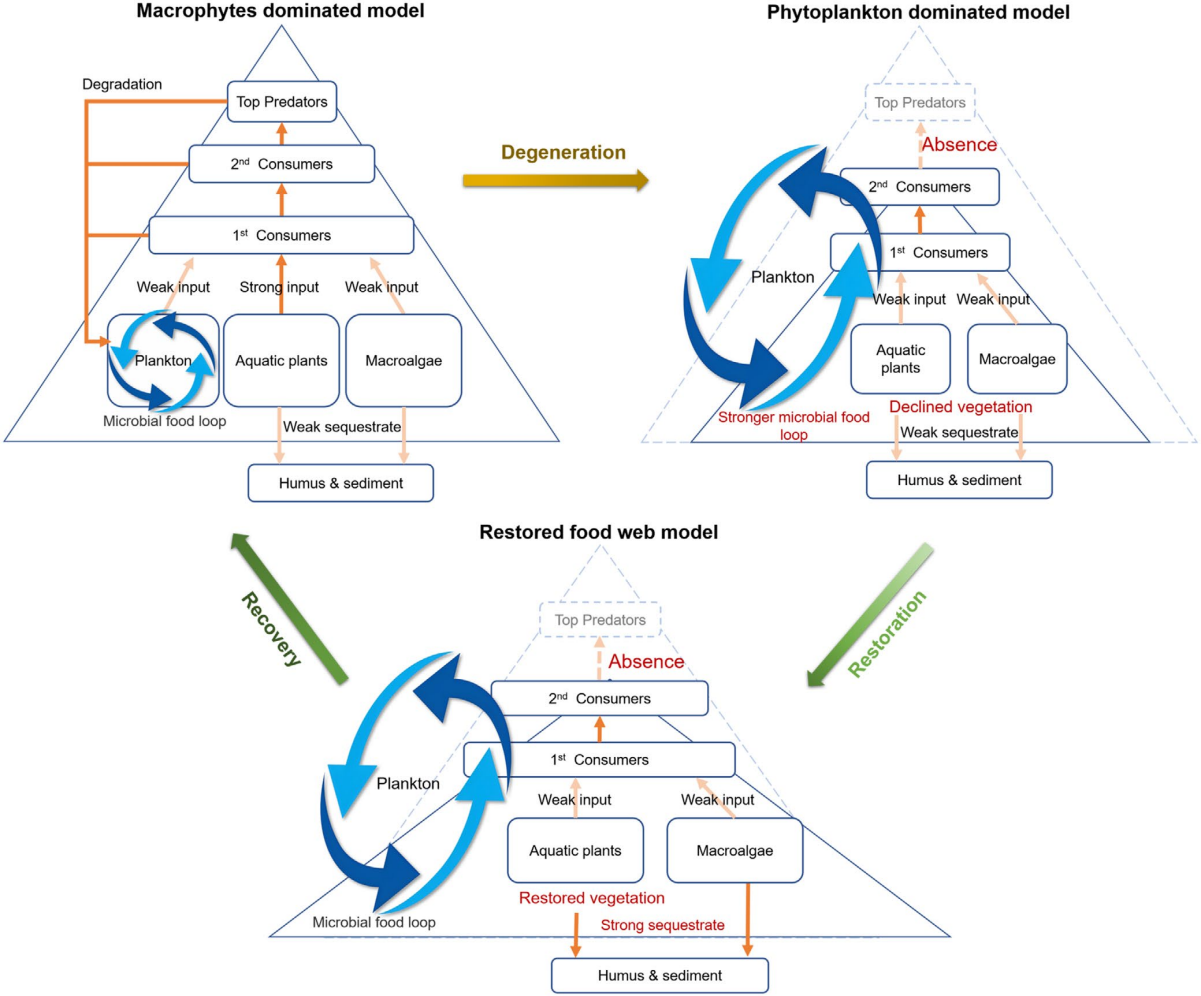
Dynamics of trophic structure within the food web of shallow freshwater lakes during degradation and restoration processes.

As shown in Figure 9, in shallow lakes, the transition from a clear‐water state dominated by macrophytes (including macroalgae and aquatic plants) to a turbid‐water state characterized by plankton dominance represents one of the most significant ecological shifts (Mao et al. 2021). Elevated nutrient inputs can lead to a decline in aquatic plants, especially submerged plants, owing to reduced water clarity caused by increased algal proliferation (Scheffer et al. 1993). In addition to nutrients, water level changes, which alter light conditions and consequently affect the growth of aquatic plants, are also a critical external driver influencing regime shifts from macrophyte dominated to phytoplankton dominated in shallow lakes (Mao et al. 2021; Zhang et al. 2017). During ecological shifts in shallow lakes, the number of top predator species declines, while the populations of intermediate and basal species remain relatively stable. Lower TLs are thought to play a more critical role in energy transfer through shortened food chains due to anthropogenic disturbances (Xu, Cai, et al. 2016). Long‐term ecological shifts lead to changes in species composition and adaptive feeding behavior among predators. Lower TL predators are pressured to increase the proportion of plankton in their diets. Meanwhile, smaller and fast‐growing fish species become dominant in total fish catches. Consequently, top predators have to feed on lower TL species, resulting in a decline in their trophic position. Higher transfer efficiencies between lower TLs and lower transfer efficiencies between upper TLs indicate reduced energy availability within a food web and might contribute to the degradation of the food web structure in shallow lake ecosystems.

This implies a potential “short circuit” in the energy flow within the planktonic food web in BYD, suggesting that a trophic cascade may occur at lower TLs as described by Xue et al. (2025). This phenomenon was associated with a higher feed conversion efficiency of species at lower TLs and negatively affected the efficiency of energy transfer through the traditional pathways, which typically involve fish and invertebrates (Xue et al. 2025). Certain plankton and invertebrate samples had higher δ^15^N values compared to some fish species, indicating that the energy transfer efficiency might be influenced at upper TLs within the food web (Figure 3).

Energy dynamics can be influenced through various mechanisms; however, shifts in trophic structure due to competitive interactions among different food sources frequently result in the predominance of one energy pathway over others (Mao et al. 2021; Xu, Cai, et al. 2016). Synchronous shifts in biotic assemblage composition and food web structure have been documented during the transition from a macrophyte‐dominated to a phytoplankton‐dominated state in previous studies. Along a eutrophication gradient in lakes across Greenland, Denmark, and the USA, increasing phytoplankton production suppressed both macrophytes and periphyton, causing local organisms to become more reliant on phytoplankton. As a result, the food web became more dependent on pelagic‐based energy flow pathways (Vadeboncoeur et al. 2003). In addition to eutrophication, elevated water levels have also been identified as a key driver of shifts in the dominance of primary producers in lake ecosystems, leading to substantial changes in trophic structure and the foundational resources that support aquatic food webs. For example, in Gucheng Lake in eastern China (Mao et al. 2021), rising water levels triggered a transition from a macrophyte‐dominated system to a phytoplankton‐dominated one—an ecological shift that induced cascading effects on food web organization. Consumers such as invertebrates and fish respond to major shifts in energy availability by stabilizing food web dynamics through their ability to utilize multiple energy pathways. They compensate for the reduced availability of macrophyte and epiphyte resources by obtaining a greater proportion of energy from the pelagic pathway (Mao et al. 2021). Once such structural and behavioral shifts are established—even if the initial stressor is mitigated—reverting to the original macrophyte‐supported trophic network may take time. Consumers may continue to exhibit pelagic feeding habits, and the balance between basal resources (macrophytes vs. phytoplankton) may require an extended period to re‐equilibrate. Therefore, our findings indicate that, despite improvements in water quality and increases in aquatic plant biomass, the recovery of trophic interactions within the BYD ecosystem, which relies heavily on plankton, will likely require more time.

## 5 Conclusion

The current trophic structure of the BYD food web was determined based on field samples and stable isotopic analysis. The results indicated that the contributions of main food sources for invertebrates and fish were as follows: plankton > aquatic plants > macroalgae. Following improvements in water quality and aquatic plant recovery due to a series of ecological restoration measures, the diversity of aquatic plant species has increased, and the TL range of fish has expanded within BYD, indicating positive progress. However, the trophic relationships among fauna remain predominantly dependent on pelagic‐based energy flow pathways, resembling a food web structure under plankton dominance. These results suggest a potential hysteresis response of the food web structure and ecosystem function to environmental restoration efforts. A more extended period will likely be required to achieve the optimal outcomes of recovery. Based on these findings, further monitoring of trophic relationship changes within the food web is recommended to better understand the ecosystem health status of shallow lakes in response to ecological restoration.

## Author Contributions

**Yajun Qiao:** conceptualization (equal), formal analysis (equal), investigation (equal), writing – original draft (equal). **Min Pang:** conceptualization (equal), data curation (lead), funding acquisition (equal), methodology (equal), validation (equal), writing – original draft (equal). **Mengyu Lv:** formal analysis (equal), investigation (equal), methodology (equal). **Tianzhe Zhu:** formal analysis (equal), investigation (equal), methodology (equal). **Wennuo Han:** formal analysis (equal), investigation (equal), methodology (equal). **Zhao Li:** formal analysis (equal), investigation (equal), methodology (equal). **Xiaolong Lin:** formal analysis (equal), investigation (equal), methodology (equal). **Peidong Su:** conceptualization (equal), project administration (equal). **Pei Qu:** conceptualization (equal), funding acquisition (equal), project administration (equal), supervision (equal), validation (equal), writing – review and editing (lead). **Chunhui Zhang:** project administration (equal), supervision (equal).

## Ethics Statement

This study was conducted with formal approval from the Ecology and Environment Bureau of Xiong'an New Area, Hebei Province, China (Permit No. HJ‐FX‐202507‐034). All animal samples were collected by local fishermen in Baiyangdian Lake in full compliance with applicable Chinese regulatory guidelines and national fisheries management policies under the supervision of the Ecology and Environment Bureau of Xiong'an New Area. Dead specimens were obtained from fishermen for use in subsequent analyses and were promptly preserved under frozen conditions during field surveys. As no live animals were handled or experiments conducted involving animal suffering, ethical approval from the university ethics committee was not required.

## Conflicts of Interest

The authors declare no conflicts of interest.

## Supporting information


**Table S1:** δ^15^N and δ^13^C stable isotope characteristics, together with TL values of field samples from Baiyangdian Lake in 2023.
**Table S2:** Descriptive information on stable isotope values of biological carbon and nitrogen collected in Baiyangdian Lake in 2018*.
**Table S3:** Descriptive information on stable isotope values of biological carbon and nitrogen collected in Baiyangdian Lake in 2019*.
**Table S4:** The TL of pelagic and benthic consumer species among three areas in Baiyangdian Lake during 2018–2019*.

## Data Availability

The data that supports the findings of this study are available in the Supporting Information of this article.
